# Control of Nanostructured Polysulfone Membrane Preparation by Phase Inversion Method

**DOI:** 10.3390/nano10122349

**Published:** 2020-11-26

**Authors:** Cristina Bărdacă Urducea, Aurelia Cristina Nechifor, Ioana Alina Dimulescu, Ovidiu Oprea, Gheorghe Nechifor, Eugenia Eftimie Totu, Ibrahim Isildak, Paul Constantin Albu, Simona Gabriela Bungău

**Affiliations:** 1 Analytical Chemistry and Environmental Engineering Department, University Politehnica of Bucharest, 011061 Bucharest, Romania; cristinabardaca@yahoo.com (C.B.U.); oanaalinadimulescu@yahoo.com (I.A.D.); gheorghe.nechifor@upb.ro (G.N.); 2 Department of Inorganic Chemistry, Physical Chemistry and Electrochemistry, University Politehnica of Bucharest, 011061 Bucharest, Romania; ovidiu73@yahoo.com; 3 Department of Bioengineering, Faculty of Chemical and Metallurgical Engineering, Yildiz Technical University, 34210 Esenler-Istanbul, Turkey; iisildak@gmail.com; 4 IFIN Horia Hulubei, Radioisotopes & Radiat Metrol Dept DRMR, 30 Reactorului Str, 023465 Magurele, Romania; plalbu@yahoo.com; 5 Faculty of Medicine and Pharmacy, University of Oradea, 1 Universităţii Str., Oradea, 410087 Bihor, Romania; simonabungau@gmail.com

**Keywords:** polysulfone membrane, phase inversion, immersion–precipitation technique, phase inversion parameters, electrochemical monitorization

## Abstract

The preparation of membranes from polymer solutions by the phase inversion method, the immersion—precipitation technique has proved since the beginning of obtaining technological membranes the most versatile and simple possibility to create polymeric membrane nanostructures. Classically, the phase inversion technique involves four essential steps: Preparation of a polymer solution in the desired solvent, the formation of the polymer solution film on a flat support, the immersion of the film in a coagulation bath containing polymer solvents, and membrane conditioning. All phase inversion stages are important for the prepared membrane’s nanostructure and have been studied in detail for more than six decades. In this paper, we explored, through an electrochemical technique, the influence of the contact time with the polymer film’s environment until the introduction into the coagulation bath. The system chosen for membrane preparation is polysulfone-dimethylformamide-aqueous ethanol solution (PSf-DMF-EW). The obtained nanostructured membranes were characterized morphologically and structurally by scanning electron microscopy (SEM) and thermal analysis (TA), and in terms of process performance through water permeation and bovine serum albumin retention (BSA). The membrane characteristics were correlated with the polymeric film exposure time to the environment until the contact with the coagulation bath, following the diagram of the electrochemical parameters provided by the electrochemical technique.

## 1. Introduction

Polymeric membranes constitute the broadest category of nanostructured filter material studied, marketed, and applied at the laboratory and industrial level [1,2,3,4].

The polymeric membranes made of polysulfone constitute an excellent filtering membrane (for micro-, ultra-, nano-, and hyper-filtration), as well as a performance matrix support for liquid membranes and macromolecular compounds or enzymes [2,5,6].

**Figure 1:**
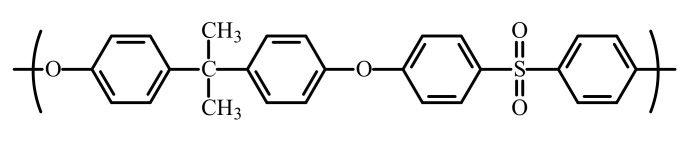
The chemical formula of the polysulfone UDEL^®^ type.

The presence of a sulfonic group characterizes polysulfone (PSf) as part of its repeating unit. In most cases is studied the polysulfone from bisphenol A and p, p′ dichloro diphenyl sulfone (Figure 1) [7]:

The polysulfone is the most cited choice for composite membrane preparation [8]. The polymer has properties that make it extremely interesting for usage as a membrane material [9]:

* good solubility in solvents commonly used in the production of membranes (dipolar aprotic solvents such as N-methyl-pyrrolidone, dimethyl-acetamide, dimethyl-formamide, dimethyl-sulfoxide);
* good resistance to acids and bases as well as to other chemical agents over a wide pH range, to oxidation, and mechanical and thermal stresses;
* the possibility to be chemically modified due to the reactive aromatic nuclei;
* relatively high glass transition temperature (T_g_ = 190 °C); and
* absence of crystallinity.

About 8–20% polysulfone solutions (commonly used to obtain membranes) have the right consistency for forming films and are adherent to various surfaces (glass, stainless steel, copper, aluminum, PVC, and teflon). Loeb and Sourirajan discovered the asymmetric membranes that could have a diffuse anisotropic or microporous anisotropic structure [1,10].

**Figure 2:**
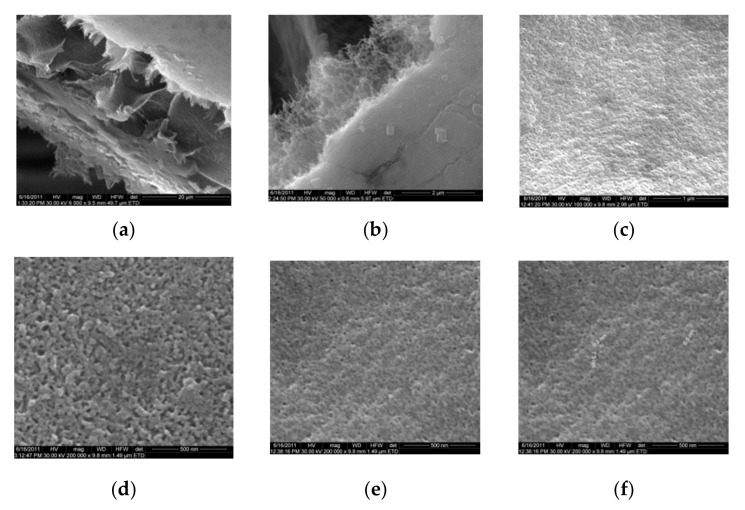
SEM images of a nanostructured polysulfone membrane (**a**)—section, (**b**)—section detail (active layer); (**c**)—surface layer and component nanoparticles; (**d**)—, (**e**)—nanostructure of microporous layer details; and (**f**)—dimension of the pores on the nanostructure surface.

Figure 2 shows the scanning electron microscopy images of a microporous, nanostructured anisotropic polysulfone membrane obtained in the research group of the Polytechnic University of Bucharest [11].

The composition influences polymeric membrane material properties obtained by phase inversion because the polymer–solvent or polymer–solute interactions affect the polymer chains’ conformation [12]. Phase inversion is the best known and currently the most applied method for obtaining membranes at the industrial level. The concept of phase inversion was introduced in the literature by Kesting [13]. It involved transforming a homogeneous polymer solution into a two-phase system: one rich in polymer and forming the continuous part of the porous membrane and another poor in polymer, which fills its structure’s pores.

The process has three main stages [14,15]:

- Dissolving the polymer in a suitable solvent or mixture of solvents;
- filming the polymer solution on a flat or tubular surface of glass, metal, teflon, or textile; and
- precipitation of the polymer by treatment with a non-solvent (inversion of the actual phase).

By phase inversion, depending on the type of polymer, the concentration of the polymer solution, the thickness of the film, and the method and conditions of precipitation, membranes are obtained for most known applications: microfiltration (MF), ultrafiltration (UF), nanofiltration (NF), and reverse osmosis (RO), with symmetrical or asymmetrical structure, in flat or tubular shape [16,17,18].

The defining step for the membrane structure is that of immersion precipitation: immersion of the polymer solution film in a coagulation solution (precipitation) where the main component is the solvent; the coagulation solution must be chosen to allow the dissolution of the solvent specific to the polymer used. The precipitation of the polymer is the result of the loss of non-solvent and penetration of the polymer film by the solvent [19,20]. This technique was first described and used successfully by Loeb and Sourirajan for the preparation of reverse osmosis membranes [10,21,22].

By phase inversion, immersion–precipitation technique, membranes are obtained from the most diverse polymers: polysulfone, nylon 6,6 [23] derived from cellulose, polycarbonate, polyphenylene oxide [24], polyoxadiazoles, polyimides [25], polyamides [26], etc.

**Figure 3:**
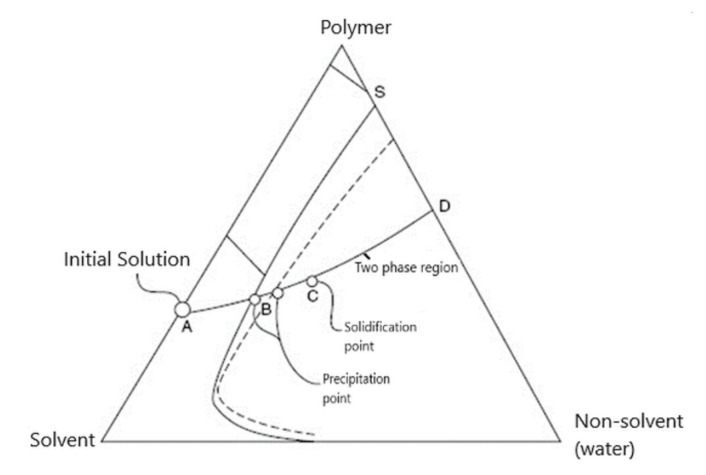
Diagram of the polymer–solvent–nonsolvent ternary system.

The following features characterize the phase inversion process:

1. The ternary system. The process involves at least one polymer phase, one solvent, and one non-solvent. The solvent and the non-solvent must be miscible (Figure 3).
2. Mass transfer. The polymer solution is subjected to a mass transfer process to enrich the polymer film in the solvent. Mass transfer begins at the interface between the polymeric film and the coagulation medium (vapor or liquid). The diffusion phenomenon governs the changes in the composition of the polymeric film.
3. The precipitation. An increase in the non-solvent concentration leads to a polymer solution thermodynamically unstable, and phase separation will occur. An essential aspect of the phase inversion is represented by a separation phenomenon in the ternary polymeric system. This phenomenon includes the equilibrium and kinetics of phase separation as the formation of membranes is a dynamic process [27,28,29].

Phase inversion occurs when the concentration of non-solvent in the polymer/solvent/non-solvent system has increased to such a level that the solution is not thermodynamically stable. Therefore, segregation of the solution will occur [30].

The structure of the membranes is determined by:

- Choice of polymer–solvent–nonsolvent system.
- Diffusion rate of the solvent outside the polymer and the non-solvent inside the polymer.

Immediately after immersion, there is a rapid depletion of the solvent in the film and relatively low penetration of the non-solvent. The concentration of the polymer at the film-bath interface increases and gelation takes place. The thin, dense gel layer that forms this way is a protective layer that will act as a resistance to the solvent’s external diffusion. Cleavage will lead to a decrease in polymer concentration and an increase in the concentration of the non-solvent. At this point, the type of splitting will be the liquid–liquid phase separation [31]. The ternary system diagram highlights the two transitions (Figure 3).

Several researchers have conducted in-depth studies on the formation of ultrafiltration membranes by applying phase inversion through precipitation immersion [24,32,33,34]. The concentrated polymer solution is first spread in a layer, film on a support, and then immersed in a non-solvent bath. Then diffusion of the non-solvent occurs, and the solvent induces phase separation, forming the porous membrane structure.

The size and distribution of membrane pores are determined by the thermodynamics [35] and kinetics [33] of the phase inversion process, being closely related to the properties of the polymer solution and the non-solvent composition [36], and the operating conditions [37].

A deep understanding of the phase inversion process is valuable to effectively adjust and control membrane structure, flow, selectivity, and clogging process [36,38,39,40,41]. Several studies have been developed on the kinetics and thermodynamics of phase inversion [42,43,44]. Tompa first determined the thermodynamics of the polymer–solvent–non-solvent system phase diagram based on the Flory–Huggins theory [45]. Moreover, Altena and Smolders investigated the binodal ternary system, considering the concentration dependence on interaction parameters [46]. 

To study the phase inversion kinetics, Cohen et al. first proposed a mass transfer model with a series of hypotheses [47], and later Reuvers et al. considered interface diffusion and friction coefficients for components [48,49]. These models considered the common hypothesis on limit conditions imposed to the equilibrium boundary established at the interface between the coagulation bath and the polymer solution [50]. They were unable to study the spinodal decomposition of the phase inversion. Therefore, Kim et al. formally introduced a mass transfer to study spinodal decomposition [51]. Akthakul et al. applied several phases and multi-component Lattice–Boltzmann (LB) models to simulate the precipitation process [52] and highlighted the membrane structure’s asymmetry: thicker top layer and thinner compact inner layer. They also found that as the interaction between the polymer and the non-solvent increased, the membrane’s morphology changed from non-porous to porous.

Finally, some research groups [12,53] focused on the computer simulation of the phase inversion process of the polymer solution from the interface to the interior by spinodal decomposition, using the Monte Carlo method. They studied the effect of polymer solution concentration and temperature on solvent and non-solvent diffusion and the formation mechanism of the porous membrane asymmetric structure.

We can conclude that, classically, the phase inversion technique involves four critical steps: preparation of a polymer solution in the desired solvent, the formation of the polymer solution film on a flat support, the immersion of the film in a coagulation bath containing polymer non-solvent, membrane conditioning. All phase inversion stages are essential for the prepared membrane’s nanostructure and were studied in detail for more than six decades.

In this paper, we study, through an electrochemical technique, the influence of the contact time with the environment of the polymer film until its introduction into the coagulation bath. The system chosen for membrane preparation is polysulfone-dimethylformamide-aqueous ethanol solution (PSf-DMF-EW).

## 2. Materials and Methods

### 2.1. Materials

The polymer used was a high viscosity BASF type (S6010—BASF, BTC Europe GmbH, Budapest, Hungary). The solvents were: N, N’dimethylformamide (DMF) (ACS puriss reagent, Sigma-Aldrich, Merck, Redox Lab Supplies Com SRL, Bucharest, Romania), and ethyl alcohol (E) (Supelco^®^, Merck, Redox Lab Supplies Com SRL, Bucharest, Romania). The ultrapure water was obtained with a Millipore RO system (MilliQ^®^ Direct 8 RO Water Purification System, Merck, Redox Lab Supplies Com SRL, Bucharest, Romania) with 18.2 μS/cm conductivity. The bovine serum albumin (BSA) (66,000 Da) used for the permeation and retention assessment was procured from Sigma-Aldrich (Merck, Redox Lab Supplies Com SRL, Bucharest, Romania).

### 2.2. Preparation of the Polymer Solution 

The determined amount of polysulfone base polymer at the desired concentration to be prepared, 12%, was gradually introduced, under stirring (magnetic stirrer, IKA^®^ C-MAG MS, from IKA^®^-Werke GmbH & Co. KG, Staufen, Germany), into the vessel containing the solvent. The mixing beaker had a lid. The mixing procedure was continued until the polymer was completely dissolved. The laboratory working conditions were: temperature 24 ± 1 °C and relative humidity of 61 ± 1%. The desired polymer solution was obtained in no more than 4 h. The obtained solution was filtered on a device equipped with a metal sieve made of stainless steel wires, with a mesh size of 20 × 20 µm. This process aims to remove solid (undissolved) impurities that negatively influence the filming process and, thus, the membrane’s structural characteristics.

The solution was subjected to the deaeration process to remove the air trapped in the dissolution process. The polymer solution’s air bubbles can lead to the appearance of discontinuities and implicitly defects in the membrane structure in the filming process. Deaeration/degassing was achieved by standing in a closed vessel for 48 h.

The degassed solutions were subsequently stored in hermetically sealed containers. The polymer solution casting was performed at room temperature (24 ± 1 °C) and humidity (61 ± 1%), using a certain amount of solution and applying it directly to a smooth glass surface (intended for thin-layer chromatography) to a thickness of 500 μm.

Immersion in the ethanol–water (EW) coagulation bath, in a 1:1 volume ratio, was performed after a predetermined time.

**Figure 4:**
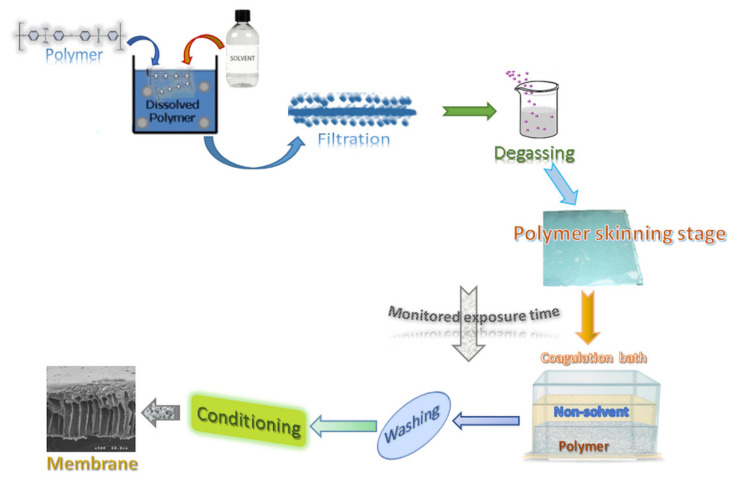
Phase inversion procedure for obtaining polysulfone membranes.

**Figure 5:**
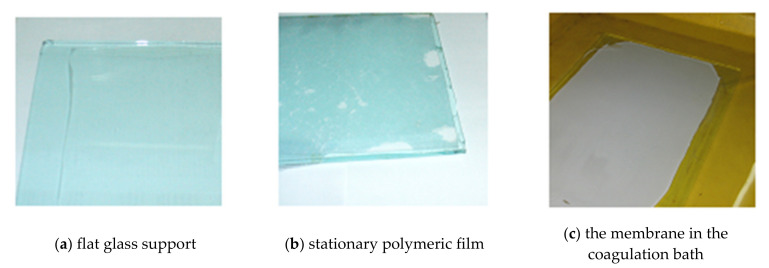
Polymeric film in the studied stage (arrow in Figure 4), between casting (**a**), stationary in the medium (**b**), and coagulation in the bath (**c**).

Figure 4 presents the primary operations carried out to obtain the membrane, highlighting the stage that is the object of the present study (Figure 5).

### 2.3. Characterization of Porous Membranes

The complete characterization of the membranes consists of determining their physico-chemical, thermomechanical, and hydrodynamic parameters [12,54,55,56,57,58]. In the followings, there are briefly presented the characterization methods applied on the membranes produced in the study.

Porosity (ε) represents the fraction of voids in the membrane structure and is calculated as the ratio between all the pores’ volume and the apparent volume of the membrane (1):

(1) $$\varepsilon =\frac{\left(V-V’\right)}{V}$$

where:

*V* = apparent membrane volume (cm^3^), and
*V′* = actual membrane volume (cm^3^).

In this paper, the porosity was determined gravimetrically, using water as a wetting agent, so that the porosity is easily determined by considering the volume of water (*V_water_*) in the membrane relative to the geometric volume of the membrane (*V_membrane_*) (2) [59]:

(2) $$\varepsilon =\frac{V_{water}}{V_{membrane}}\;\cdot 100$$

The permeability of liquids (water) expressed as their hydrodynamic flow when applying a pressure gradient offers the possibility to determine the average pore radius applying the Hagen–Poiseuille equation, adapted by Vellicangil and Howell for asymmetric membranes (3) [60]:

(3) $$J=\frac{\varepsilon \cdot r\cdot \Delta p}{3\cdot \pi \cdot \eta }$$

where:

*J* = hydrodynamic flow;
*r* = average pore radius;
Δ*p* = pressure difference when measuring the flow *J*, and
η = viscosity of the reference liquid (water).

The membranes performance in terms of separation capacity and flow characteristics are given by two fundamental parameters: Flow and selectivity.

The flow (*J*) is generally defined as the volume of fluid (water, in this case) that passes through the membrane in the unit of time per unit area (4) [20,61,62]:

(4) $$J=\;\frac{V}{S\;\cdot t}$$

where:

*J* = flow (L/m^2^h);
*V* = the volume of fluid passed through the membrane (L);
*S* = membrane surface (m^2^), and
*t* = time (h).

The decision to use a membrane in a particular process is based primarily on the standardized feature called “normalized distilled water flow”. It is determined from the ratio of the volume of distilled water flowing through a membrane in a given time to a certain pressure difference (5):

(5) $$J=\;\frac{V}{S\;\cdot t\;\cdot \;\Delta p}$$

where:

*J* = flow (L/m^2^h bar);
*V* = distilled water volume (L);
*S* = membrane surface (m^2^);
*t* = time (h), and
Δ*p* = pressure difference (bar).

Usually, the membranes are tested to determine the normalized water flow at a pressure difference of 1 bar.

The selectivity or retention (*R*) of membranes represents their ability to retain on their surface a single component of a mixture (solution). It is expressed in the form of the degree of retention (*R*), determined according to Formula (6):

(6) $$R=\;\frac{c_{f}-\;c_{p}}{c_{f}}=1-\;\frac{c_{p}}{c_{f}}$$

where:

*c_f_* = solute concentration in the supply fluid (% mass, g/L, mols/L);
*c_p_* = concentration of solute in the permeate (% mass, g/L, mols/L).

In our case, we used bovine serum albumin (BSA, 69 kDa, Merck, Redox Lab Supplies Com SRL, Bucharest, Romania).

### 2.4. Preparation of Membrane Samples

**Figure 6:**
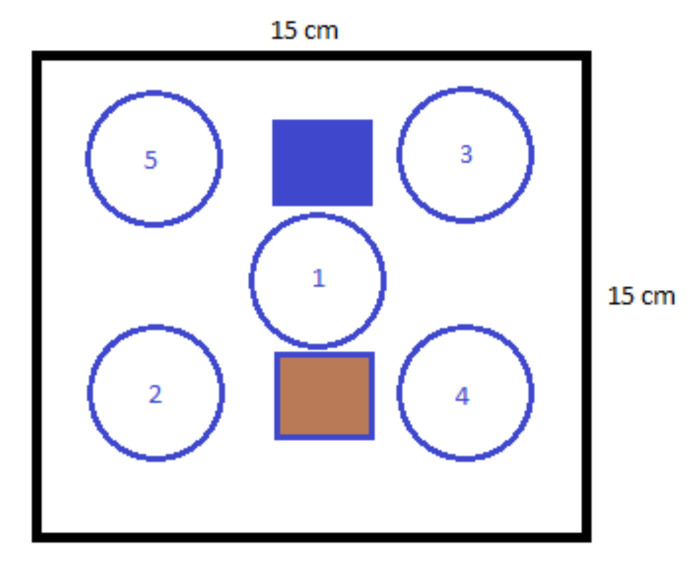
The sampling from the membrane under analysis—circular samples: 1 to 5 and the brown and blue rectangles.

After coagulation of the polymer film, the membrane was washed and dried in a vacuum oven for 48 h at 60 °C. Then, the central part of the membrane was cut to the size of 15 cm × 15 cm. Afterwards, five samples as discs with a diameter of 5 cm and two rectangles (5 cm × 5 cm) were obtained (Figure 6).

The porosity, water flows, and BSA retentions were determined for each membrane type using the five samples. For thermal analysis, the brown sample was used, and for electron microscopy, the blue sample.

### 2.5. Structural, Thermal, and Electrochemical Investigations

Electron microscopy offers the possibility to visualize the porous structure of membranes [45]. The upper surface and cross-section analysis give a clear picture of the general structure of the membrane. Thus, it highlights the thickness of the active and macroporous layer, and estimates can be made of the porosity and pore distribution according to the radius size. SEM investigations were performed on a Hitachi S4500 system (Hitachi High-Technologies Europe GmbH, Germany).

Thermal analysis followed both weight loss (TG) and thermal effects (DSC). The importance of the thermal analysis in the polymeric membranes is well-established [25,58]. The polymeric specimens under study were thermally analyzed using a Netzsch Thermal Analyzer (NETZSCH-Gerätebau GmbH, Germany). The experiments were run in nitrogen atmosphere at 10 °C/min heating rate, covering a temperature range from 25 °C to 500 °C.

Electrochemical monitoring of the polymeric film between skinning (top skin formation) and coagulation was performed using an electrochemical probe on which the polysulfone solution film was deposited.

A complex electrochemical system as the dielectric analyzer—DEA 288—Netzsch (NETZSCH-Gerätebau GmbH, Selb, Germany) using a specific sensor (Coated Tool Mountable Comb—NETZSCH-Gerätebau GmbH, Germany) allowed advanced electrochemical studies on polymeric samples. The casted membrane film thicknesses were about 3 mm. The frequency applied for measurements was 10 Hz.

## 3. Results

### 3.1. Evolution of Electrochemical Parameters

**Figure 7:**
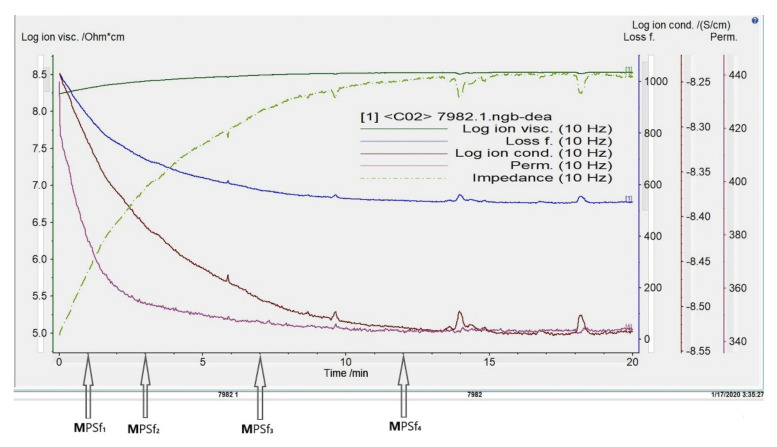
Diagram of electrochemical parameters vs. time and the moments when each film was introduced into the coagulation bath to obtain the analyzed membranes (MPSf1, MPSf2, MPSf3, and MPSf4).

The evolution of the polymeric film’s main electrochemical parameters until the immersion in the coagulation bath is presented in the diagram from Figure 7. The main electrochemical parameters that were followed up: ionic viscosity, ionic conductivity, loss factor, permittivity, or impedance, allowed to draw the frame for the PSf film evolution when left it in contact with the environment. Usually, ion viscosity and loss factor could show the progress of a polymeric system at ambient temperature.

On the diagram presented in Figure 7 it could be observed as an initially quite reduced loss factor increases slightly with the exposure time correlated with the viscosity decrease. It is reasonable to consider that the minimum value for the viscosity (occurring before gelation) corresponds to the loss maximum value.

The diagram shown in Figure 7 indicates the time after which the polymeric film formed on the glass support and exposed to the environment is immersed in the coagulation bath. Each film is left in contact with the environment (atmosphere) 1, 3, 7, and, respectively, 12 min after which it is immersed in the coagulation bath, forming the membranes MPSf1, MPSf2, MPSf3, and MPSf4.

### 3.2. Scanning Electron Microscopy

For the morphological study by scanning electron microscopy, a sample was fractured in liquid nitrogen, and the surface, posterior surface, and membrane section were visualized (Table 1). Table 1 presents the morphology of the surface and the posterior surface porosity. Also, there are presented the average thickness of the membranes and their porosity (ε%).

### 3.3. Thermal Analysis

**Figure 8:**
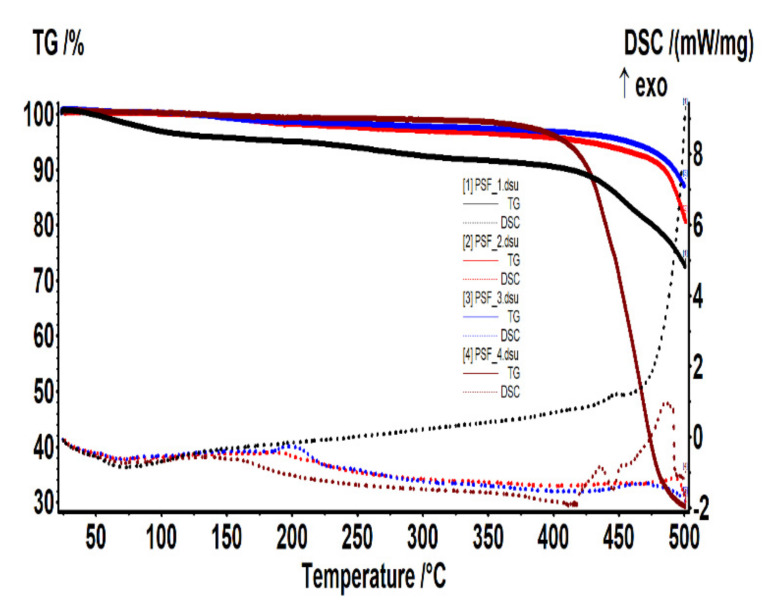
The set of thermal analysis data for the four membranes.

Figure 8 shows (as assembly) the thermal analysis performed on the sample cut from each of the four membrane types.

The centralized data show both the weight loss (TG) and the thermal effects (DSC) for the MPSf1, MPSf2, MPSf3, and MPSf4 membranes at 10 °C/min heating rate.

### 3.4. Determination of Water Flows and Retention of Bovine Serum Albumin (BSA)

Process performance was checked for five samples from each of the four prepared membranes. The experiments were performed on a Sartorius device with parallel filter modules to compare and measure flows and retention, respectively.

Experiments performed with pure water at a transmembrane pressure of 2 bar allowed to establish the normalized flow (*J*) [46]. For selectivity (*R*), BSA was retained from a solution of 100 mg/L [47] (Table 2).

## 4. Discussion

The concept of phase inversion introduced by Kesting [29,30] in the 1970s can be summarized as follows: a homogeneous polymer solution is transformed into a two-phase system in which the solid phase is represented by the polymer that forms the membrane. In contrast, the liquid phase, in a tiny proportion, in the polymer structure will then lead to the formation of the pores. Phase inversion by precipitation immersion remains a crucial research topic as the details of the technological process of obtaining membranes (Figure 4), although relatively simple, involves multiple secondary operating parameters. Of these, in this study we focused on the polymer film’s standing time in the environment (atmosphere) before entering the coagulation bath. The importance of ionic conductivity in obtaining polymeric membranes has been underlined in some of our previous work [63].

Considering that there are *n_PSf_* ions of the polymer (*PSf*) per unit volume, with *q_PSf_* charge magnitude and ionic mobility *m_PSf_*, the ionic conductivity is expressed by:

(7) $$\sigma =\;\sum_{PSf}n_{PSf}\cdot \;q_{PSf}\cdot m_{PSf}.$$

The relationship between the mobility of the ion, *m_PSf_*, and the characteristics of the polymer could be described through a simple Stoke model [64] that follows the velocity of a small particle (spherical with a radius *r_PSf_*) in a homogeneous fluid material with a viscosity η*_PSf_* when a force (i.e., electrical *q_PSf_E*) acts on it.

(8) $$F_{PSf}=q_{PSf}E=\left(6\cdot \pi \cdot r_{PSf}\cdot \eta_{PSf}\cdot v_{PSf}\right).$$

When a specific current *E* is applied, then the velocity of each considered polymeric ion is:

(9) $$v_{PSf}=\;m_{PSf}\cdot E$$

Therefore, the mobility of the ions under the conditions mentioned above is given by:

(10) $$m_{PSf}=\;q_{PSf}/\left(6\cdot \pi \cdot r_{PSf}\cdot \eta_{PSf}\right).$$

Under such simplified model, the ionic conductivity becomes:

(11) $$\sigma =\sum_{PSf}(\frac{n_{PSf}\cdot q_{PSf}^{2}}{6\cdot \pi \cdot r_{PSf}\cdot \eta_{PSf}}).$$

Therefore, it results that the ionic conductivity varies with (1/η). However, it should be mentioned that the above considerations apply until the gelation process. The mobility m_PSf_, and the viscosity depend on the mobility of the polymer segments. Consequently, before the gelation process, both viscosity and ionic conductivity behave similarly. After the polymeric network is formed (at gelation), the viscosity tends to infinite. However, ionic conductivity is still finite as the polymer segments maintain their mobility to a certain extent. 

The presence of the polar molecular polymeric segments influences the permittivity; consequently, the recorded permittivity decreases with time exposure increasing. Performing the measurements at various frequencies, it was observed that at low frequencies a dipolar alignment was possible and reflected in ion conductivity that could be related to polymer chains mobility. The first inflection point (given by the 1st derivative) on the ionic viscosity curve (logη*_PSf_* = 8.28 Ω.cm) occurred at 1.1 min. This was the reason for considering as the first exposure time 1 min. Although, the variation of the ionic conductivity, in accordance with the ionic viscosity, was into a narrow range 10^−8.25^ S/cm and 10^−8.52^ S/cm, it allowed to differentiate between the specific exposure times when the polysulfone underwent membrane preforming processes. Thus, the following inflection points recorded at 3.1 min, 6.8 min, and 12.0 min gave a hint regarding the choosing of the suitable exposure times. After 18 min, when the recorded electrochemical curves are flat at logη*_PSf_* = 8.52 Ω.cm, respectively, log*σ* = −8.52 S/cm, and the permittivity is 3.46, the polysulfone membrane preformation ended.

**Figure 9:**
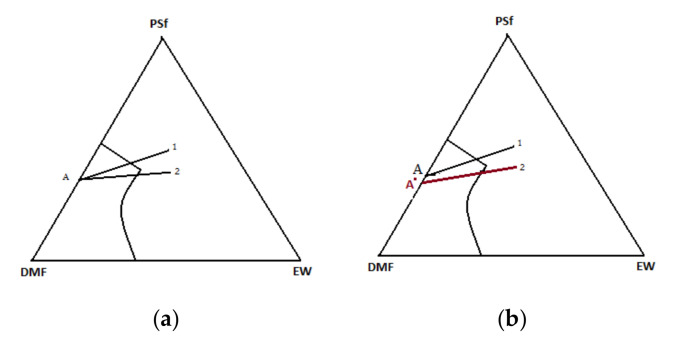
The schematic composition route for the active layer (1) and the porous layer (2) of the polymeric film starting from the initial composition A: according to the theory—(**a**) and according to this work—(**b**).

It is almost unanimously accepted that during the precipitation immersion process, the membrane is formed by extracting the solvent into the non-solvent (the two solvents being miscible). Cohen [47] and Smolders [48] suggested that a gelling process forms the active layer. In contrast, the formation of the porous support layer results from a liquid–liquid phase separation that generates centers of nucleation and an increase in the polymer structure (Figure 9). The determining factor for the type of phase separation at any point in the solution is the local polymer concentration during precipitation. Immediately after immersion, the solvent’s rapid migration around the polymer macromolecular chains takes place simultaneously with a slight penetration of the non-solvent. This is reflected in the polymer concentration increase at the interface between the film and the coagulation bath, thus starting the formation of the gel layer. The gel formed representing the active layer (in an incipient form) of the membrane will begin to act as a barrier on the remaining non-solvent, thus leading to the solidification of the gel (increasing the concentration of non-solvent and decreasing the concentration of solvent). Thus, Figure 9 explains the path taken in forming the active layer and, respectively, the microporous substrate of the asymmetric membrane obtained by phase inversion. 

**Figure 10:**
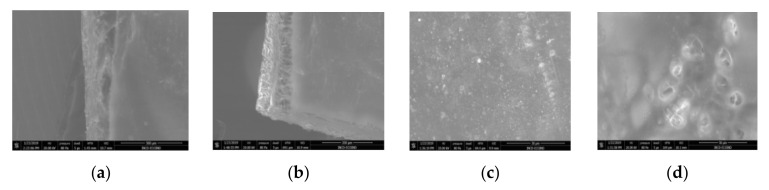
Polysulfone membrane (independent validation analysis): (**a**)—Upper active layer (section), (**b**)—bottom active layer (section), (**c**)—surface and (**d**)—back surface (bottom surface).

Figure 10 shows the SEM microscopy only for one of the prepared membranes (MPSf2) performed at ECOIND (an independent laboratory facility) as a requirement to validate the morphology of the obtained membranes. Both active layers (upper and bottom) highlighted in section (Figure 10a,b), as well as the aspect of the surfaces (upper and bottom) (Figure 10c,d) confirm the results obtained in the experimental part—see Table 1.

**Figure 11:**
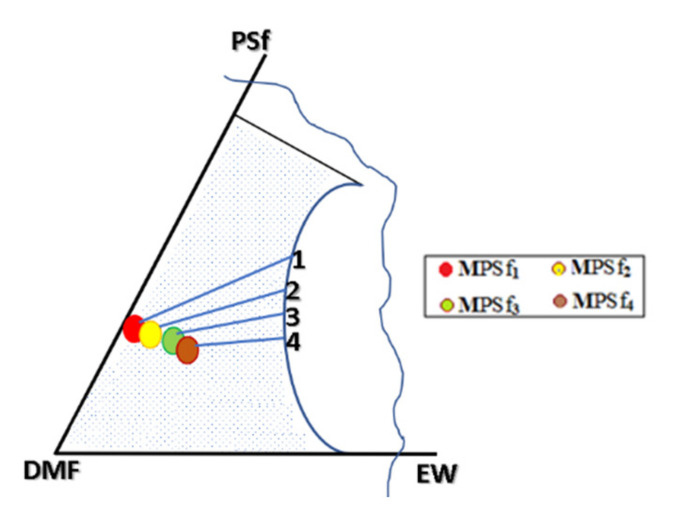
The schematic composition route of the polymeric film starting from the composition determined by the contact with the environment at times of 1 min (MPSf1), 3 min (MPSf2), 7 min (MPSf3), and 12 min (MPSf4).

In this paper, for the PSf-DMF-EW ternary system in discussion, it is studied the position of point A (Figure 9) from which the coagulation started. Thus, the ternary system’s departure from point A (homogeneous solution of polysulfone in DMF), both for forming the active layer and the microporous substrate (Figure 9a), is obviously forced. This statement is justified because, after forming the active layer, a quantity of polymer is removed from the system. The macroporous layer’s coagulation begins in a solution of lower concentration (point A′ on Figure 9b) due to the fact that the polysulfone film in dimethylformamide practically never starts from point A where the PSF-DMF side is from a homogeneous system. Much closer to reality, and as this work experiments prove it, the starting point in coagulation is inside the diagram, so the coagulation in the bath starts from a ternary system as highlighted in Figure 11.

**Figure 12:**
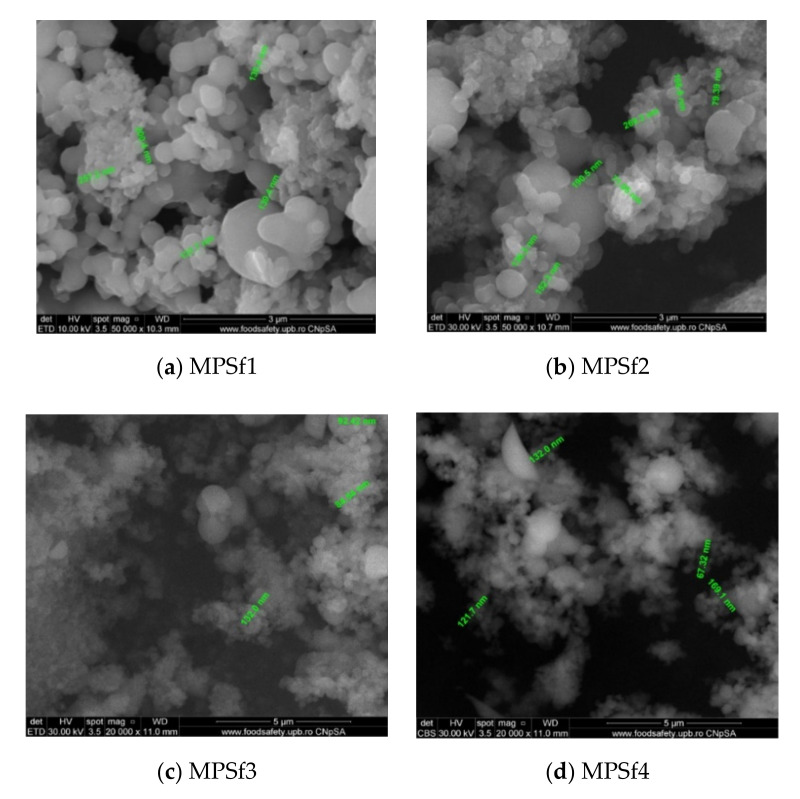
Morphology and dimensions of micro- and nanoparticles in the membrane section.

Evidently, for graphic reasons, the left-down corner position of the ternary diagram was exaggerated—Figure 11. Still, it complies with the physical reality in that the polymeric film is penetrated by chemical species from the atmosphere (water vapor and carbon dioxide) that determine the beginning of phase inversion, the transition to a biphasic system (dispersion of polymeric nanoparticles in polysulfone solution with a slightly lower concentration—Figure 11). In this way, asymmetric membranes with different characteristics are obtained. Although, the asymmetric aspect is identical for all the obtained membranes, the surface morphology is different (Table 1), resulting in smaller and smaller pore sizes when the exposure time to environment increases. The pore size is determined by the dimension of the nanoparticles (highlighted in Figure 12) that generate them by intercalation, and it decreases in order: MPSf1 > MPSf2 > MPSf3 > MPSf4.

**Figure 13:**
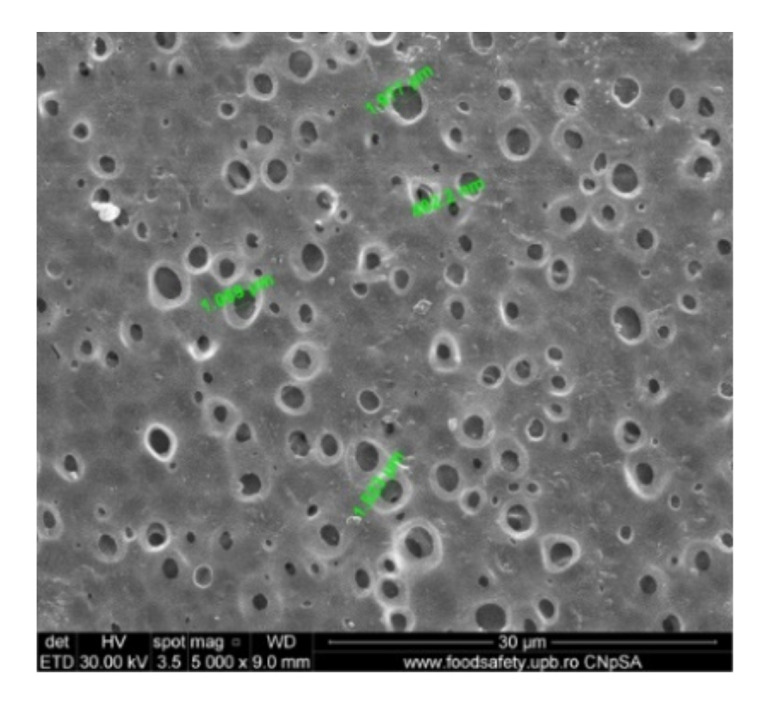
Illustration of the pore size on the posterior surface in the SEM image obtained for MPSf1.

Of course, the pore size of open micropores on the posterior surface varies in the same direction: MPSf1 > MPSf2 > MPSf3 > MPSf4 and it was determined by measuring all pores on the image obtained by SEM microscopy as indicated. In Figure 13 it is presented the pore size assessment for the MPSf1 membrane. 

As known, the characterization of the surface morphology means the visualization of a tiny area. The information can be relative, but the macroscopic determinations, such as the porosity of the membranes determined gravimetrically by watering could give a solid support. The porosity data obtained (Table 1) confirmed the previous observation, respectively, that the porosity decreases as εMPSf1 > εMPSf2 > εMPSf3 > εMPSf4.

The thermal characterization, which theoretically would have generated identical results for the four membranes, provided interesting information that confirms that the four membranes have different thicknesses and that they are nanostructured, which (if conditioned) often have a remnant adsorbed content.

**Figure 14:**
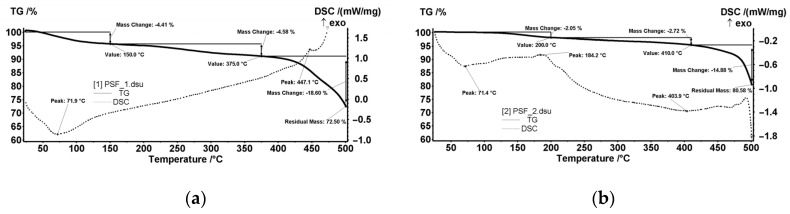
Weight loss (TG) and thermal effects (DSC) diagrams for MPSf1 (**a**) and MPSf2 (**b**).

The MPSf1 sample (Figure 14a) lost 4.41% of the initial mass up to 150 °C with an endothermic effect having a minimum of 71.9 °C. Most likely, the sample lost weakly bound solvent (water) molecules. Between 150–375 °C, the sample mass decreased with 4.58% without a clear contoured effect on the DSC curve. This could be assigned to the exothermic effects that could overlap with endotherms of about the same intensity, so only the resultant effect is visible—it would mean a slight decomposition, accompanied by partial oxidation.

After 375 °C, the sample started to suffer an accelerated oxidative degradation, up to 500 °C, losing 18.60% of the initial mass. A first exothermic effect was observed at 447.1 °C, the second exothermic effect having the maximum after 500 °C. The residual mass at 500 °C was 72.50%.

The MPSf2 sample (Figure 14b) lost 2.05% of the mass between room temperature (RT) and 200 °C. The process was accompanied by an endothermic effect with a minimum at 71.4 °C, which indicated the loss of some solvent/water molecules. Towards the end of the interval, a peak could be observed at 184.2 °C, probably from a weak oxidation.

Between 200–410 °C, the sample lost 2.72% of the initial mass. The process was accompanied by a weak endothermic effect, broad, which indicated a slight decomposition. After 410 °C, the sample started to decompose faster, up to 500 °C, losing 14.88% of the mass. The thermal effect was endothermic, which indicated a predominant decomposition process (unlike the one from MPSF1 where oxidation predominates). The residual mass at 500 °C was 80.58%.

**Figure 15:**
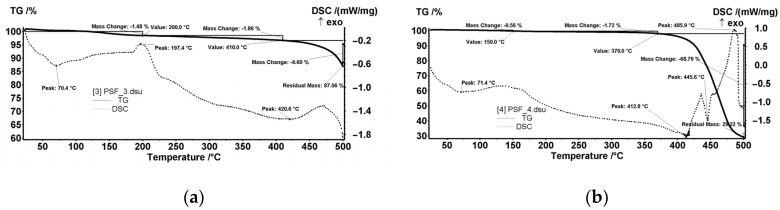
TG and DSC diagrams MPSf3 (**a**) and MPSf4 (**b**).

The MPSf3 sample (Figure 15a) lost 1.48% of the mass between RT-200 °C. An endothermic effect with a minimum at 70.4 °C accompanies this process that indicated the loss of some solvent/water molecules. Towards the end of the interval, an exothermic peak was observed at 197.4 °C, probably from weak oxidation. Between 200–410 °C, the sample recorded 1.86% decrease of the initial mass. The process was accompanied by a weak endothermic effect, broad, which indicates a slight decomposition. After 410 °C, the sample started to decompose faster, up to 500 °C, losing 9.60% of the mass. The thermal effect is endothermic, which indicated a predominant decomposition process. The residual mass at 500 °C was 87.06%.

For the MPSf4 sample (Figure 15b), in the RT-150 °C interval, the sample lost 0.56% of the initial mass, the endothermic process having the minimum at 71.4 °C. Between 150–370 °C, the sample mass decreased with 1.72%. After 370 °C, the decomposition of the sample occurred, the mass loss being of 68.79% up to 500 °C. The DSC curve showed two endothermic effects (corresponding to decompositions) with minimums at 412.8 and 445.6 °C. An exothermic process with a maximum at 485.9 °C, indicating an oxidation process, followed the previous thermic effects.

The residual mass at 500 °C was 29.22%, which could be explained by the polymer and excess solvents’ combustion, which also generated the observed thermal effect.

Two hypotheses might explain the results of the thermal analysis:

- The thickness of the membranes may indirectly influence this process. For analysis, the crucible usually contains at least a 4–5 mg sample. It means to use several round cut membranes (disks), one on top of the other. The membrane disks’ thickness conditions the number of disks used: 2–3 or 4–6 disks if it is too thin. The oxygen access (easier or difficult) towards the sample depends on its thickness. Consequently, depending on how many membrane disks are in the crucible, the decomposition, or oxidation processes at high temperatures may predominate.
- The size of the polymeric nanoparticles that form the membranes and the fact that they superficially retain the liquid components of the thermal system influence the objectivity of results.

Both hypotheses correlate with the experimental data observed by electron microscopy, porosity, and the geometric (micrometric) thickness of the membranes (Table 1).

To objectively state the decisive influence of the exposure time on the polysulfone solution film before immersion into the coagulation bath (as part of the membranes obtaining process by precipitation immersion), we performed the classical tests of pure water permeation and retention of a standard protein (BSA) (Table 2).

The results show that the normalized solvent flows (J) decrease in the order:

J_MPSf1_ > J_MPSf2_ > J_MPSf3_ > J_MPSf4_

which correlates very well with the thickness and porosity of the membranes. 

The retention values (R) generally confirm the findings:

R_MPSf3_ > R_MPSf4_ > R_MPSf2_ > R_MPSf1_

The slight decrease in retention in the case of the MPSf4 membrane is a consequence of the membrane collapse due to the solvents remaining in a larger quantity on the particle surface, a fact observed in the deviation of other determining characteristics.

## 5. Conclusions

Obtaining membranes by the phase inversion method, the precipitation immersion technique, remains one of the most accessible for obtaining asymmetric anisotropic membranes.

Although the precipitation immersion technique is simple, it consists of forming a polymer solution film, extending it on support, and immersing it in the coagulation bath. It has multiple operational parameters: polymer solution concentration, solvent nature, non-solvent nature, and type of post-formation treatment.

In this context, although it is often specified, for the time elapsed between the formation of the polymer solution film and its contact with the coagulation bath no method has been developed to track its influence on the formation of the asymmetric membrane.

This paper presents an electrochemical technique for tracking the evolution of the polymer solution film. By choosing the contact time, it was possible to establish its influence on the membrane formed by subsequent coagulation.

To highlight this process, four representative times were chosen based on the electrochemical parameters diagram, obtaining four different membranes: MPSf1; MPSf2; MPSf3, and MPSf4.

Through complementary membrane analysis techniques as scanning electron microscopy, thermal analysis, water permeation, bovine serum albumin retention, but also porosity and thickness, it was established that the process performances in terms of fluxes and retention of the membranes increase in the order: MPSf1 > MPSf12 > MPSf3 < MPSf4.

Our work proved that to obtain membranes with desired performances, it is necessary to establish an optimal stationary time for contact with the environment, which could be determined from the specific behavior of electrochemical parameters for the polymer solution film. Simultaneously, the maximum contact time with the environment can be established so that to avoid obtaining membranes with inferior characteristics.

## Figures and Tables

**Table 1:** Morphology of the surface, posterior surface porosity, average thickness, and porosity of the membranes obtained.

Membrane *	Surface	Bottom	L (µm)	ε (%)
MPSf1	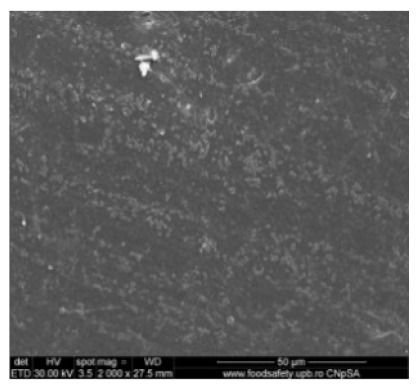	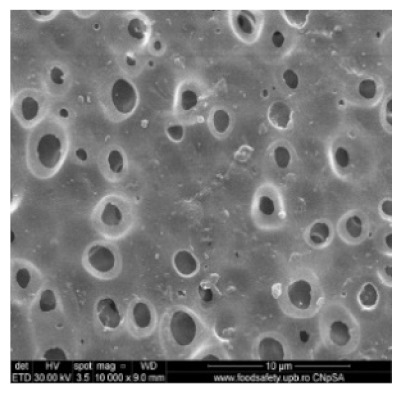	126 ± 2	72 ± 3
MPSf2	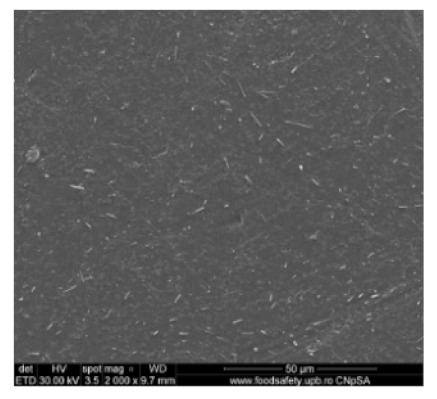	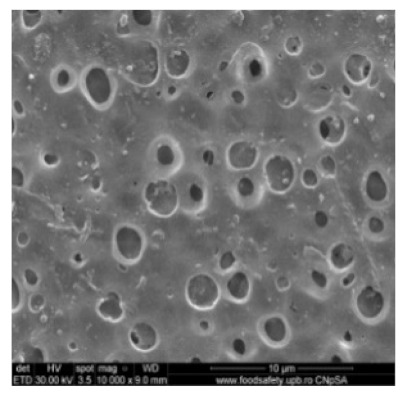	124 ± 2	69 ± 3
MPSf3	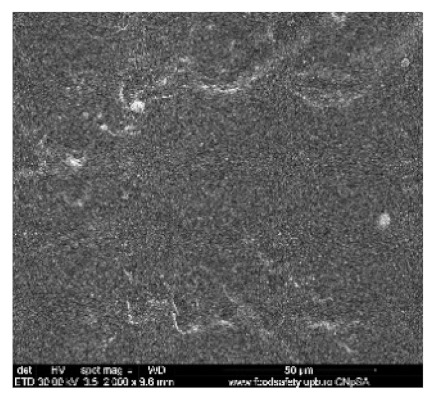	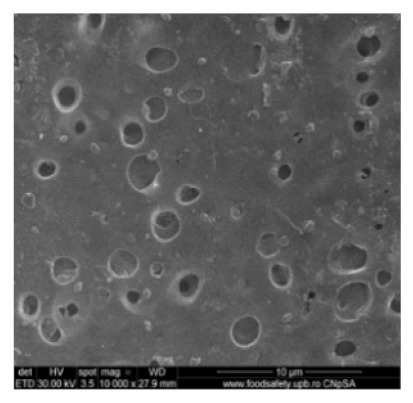	123 ± 2	67 ± 3
MPSf4	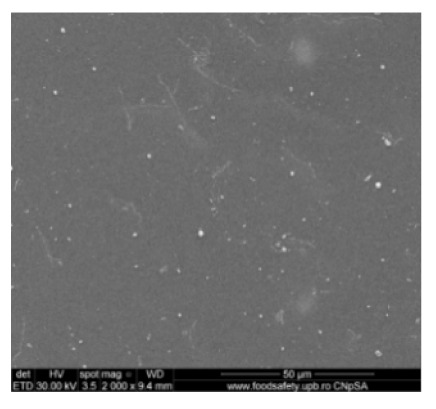	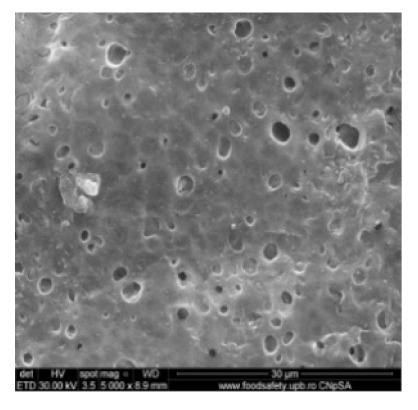	117 ± 6	63 ± 5

* The membranes MPSf1, MPSf2, MPSf3, and MPSf4 were those obtained after different exposure times to environment prior to their immesion into the coagulation bath, respectively: 1 min, 3 min, 7 min, and 12 min.

**Table 2:** Normalized flow (*J*) and Selectivity (*R*) for the prepared membranes.

Membrane Type	Normalized Flow, J (L/m^2^·h·bar)	Retention, R (%)
MPSf_1_	73 ± 2	75 ± 3
MPSf_2_	61 ± 2	82 ± 3
MPSf_3_	54 ± 2	93 ± 3
MPSf_4_	51 ± 2	84 ± 3
